# A nucleoside transporter on the mitochondria of *T. gondii* is essential for maintaining normal growth of the parasite

**DOI:** 10.1186/s13071-025-07054-w

**Published:** 2025-10-21

**Authors:** Jiahui Qian, Liyu Guo, Yilian Yang, Zhengming He, Mingfeng He, Chengjie Chen, Yifan Luo, Jiayin Xu, Senyang Li, Rui Fang

**Affiliations:** 1 State Key Laboratory of Agricultural Microbiology, College of Veterinary Medicine, Huazhong Agricultural University, Wuhan, 430070 Hubei People’s Republic of China; 2 College of Veterinary Medicine, Henan Agricultural University, No. 218 Longzihu University Area, Zhengdong New District, Zhengzhou, 450046 China

**Keywords:** *T. gondii*, Mitochondrial, Nucleoside transporter

## Abstract

**Background:**

*Toxoplasma gondii* invades almost all nucleated cells of warm-blooded animals, but *T. gondii* lacks the ability to synthesize purines; therefore, it must scavenge purines from host cells to fuel its proliferation and propagation. Through exogenous expression, the adenosine transporter *Tg*AT1 has been validated for its ability to transport both oxypurine nucleosides and nucleobases across the parasite plasma membrane, although its affinity for the substrate is low. Further studies have shown that *T. gondii* also has a high-affinity purine and pyrimidine nucleoside transport system (*Tg*AT2), but this protein has not been identified thus far.

**Methods:**

Here, we identified three novel nucleoside transporters in *T. gondii* by homology alignment. Using immunofluorescence staining, we found that *Tg*NT1, *Tg*NT2, and *Tg*NT3 are localized to the mitochondria, plasma membrane, and endoplasmic reticulum (ER), respectively. We also performed conditional knockout of *Tg*NT1 and direct knockout of *Tg*NT2/*Tg*NT3 using CRISPR/Cas9 technology.

**Results:**

*Tg*NT1 is crucial for the in vitro growth and proliferation of *T. gondii*, whereas the other two genes are dispensable. Conditional depletion of *Tg*NT1 impairs multiple metabolic pathways in both the mitochondria and cells of the parasite, with the most significant changes manifesting in the levels of various nucleosides. Carbon metabolism is also affected, as evidenced by alterations in metabolite levels within both the electron transport chain and the tricarboxylic acid cycle. The impairment of adhesion and invasion functions appears to be strongly associated with a reduction in the content of the initial lipid involved in glycosylphosphatidylinositol (GPI)-anchored lipid modification, which may underlie the inhibition of *T. gondii*’s invasive capacity. Notably, *Tg*NT1 is the first nucleoside transporter protein that is localized in the mitochondria of *T. gondii*. Given that *Tg*NT1 has no homologous proteins in mammals, it holds promise as a potential drug target.

**Conclusions:**

*Tg*NT1, a nucleoside transporter located in the mitochondria of *T. gondii*, is essential for maintaining the normal growth of the parasite.

**Graphical Abstract:**

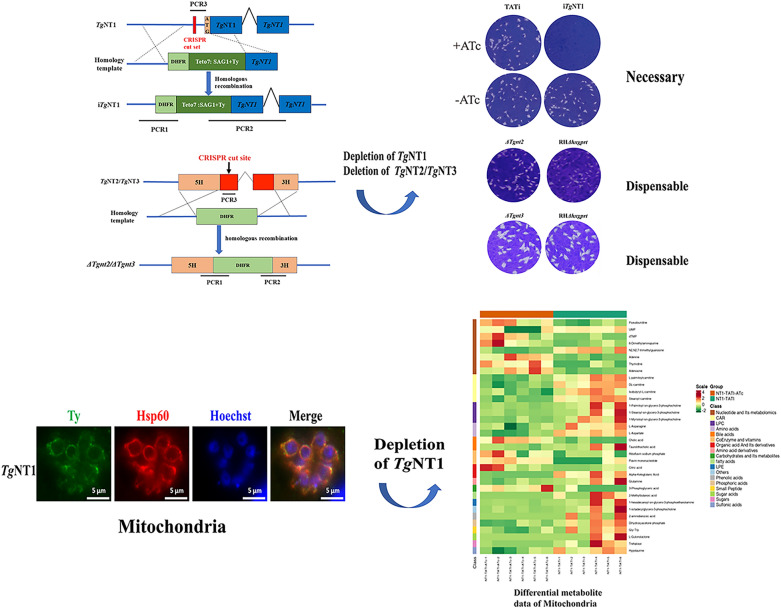

**Supplementary Information:**

The online version contains supplementary material available at 10.1186/s13071-025-07054-w.

## Background

*Toxoplasma gondii* is an obligate intracellular parasitic protozoan that infects more than two billion people worldwide [1, 2]. The overall incidence rate of *T. gondii* in livestock and poultry is 28.3% [3]. Infection with *T. gondii* does not cause serious symptoms in normal populations, but the parasite can form cysts and persist in multiple organs. However, infection with *T. gondii* in patients with acquired immunodeficiency syndrome (AIDS) and organ transplant recipients is fatal [4]. Pregnant women infected with *T. gondii* for the first time may experience symptoms such as miscarriage and stillbirth [1, 5].

The current mainstream treatment for toxoplasmosis involves a combination of pyrimethamine and sulfadiazine, with folinic acid administered concomitantly to mitigate toxicity. Importantly, these two drugs are only effective against the tachyzoite stage of *T.gondii* and fail to eliminate cysts residing in muscle and brain tissues [6, 7]. Research into anti-toxoplasmic drugs targeting *T. gondii* is ongoing, with a pressing need to identify safe, nontoxic agents that not only eliminate tachyzoites but also effectively eradicate tissue cysts [8]. In recent years, nucleoside analogs have emerged as pivotal agents in combating *T. gondii*. A range of such analogs, including 6-benzylthioinosine derivatives and 5-fluoropyrimidines, have demonstrated greater efficacy and improved safety profiles against *T. gondii* compared with conventional chemotherapeutic agents [8, 9]. For these nucleoside analogs to reach their site of action, they must be transported by the nucleoside transporters of *T. gondii*. Thus, in-depth research on *T. gondii* nucleoside transporters also provides a theoretical foundation for the development of novel anti-*T. gondii* nucleoside analogs.

In *T. gondii*, *Tg*AT1 is currently the only characterized nucleoside transporter. *Tg*AT2, though it is considered to exhibit high-affinity adenosine/inosine transport activity, remains unannotated to date [10–12]. In this study, we identified three putative nucleoside transporters in *T. gondii* by homology alignment. Immunofluorescence staining revealed their distinct subcellular localizations: one in the mitochondria, another in the plasma membrane, and the third in the endoplasmic reticulum (ER) of the parasite. Among these three nucleoside transporters, only *Tg*NT1 was found to be essential—its depletion led to a significant inhibition of in vitro replication and growth. Metabolomic analysis of this depletion strain further confirmed that mitochondrion-localized nucleoside transporters are critical for the in vitro metabolism of *T. gondii*.

## Methods

### Parasite strains and cell culture

The parent strains included RH*Δku80*, RH*Δhxgprt*, and RH-TATi. These strains were routinely passaged in vitro in monolayers of human foreskin fibroblasts (HFF) at 37 °C in 5% CO_2_ as previously described [13–15]. HFF cells were grown in Dulbecco’s modified Eagle medium (DMEM; GIBCO) supplemented with 10% fetal bovine serum (FBS), 2 mM l-glutamine, and 1% penicillin–streptomycin, and the *T. gondii* strains were grown in culture medium supplemented with 2% FBS.

### Plasmid and mutant strain construction

CRISPR/Cas9 plasmids were used to construct the different mutant strains. All the CRISPR/Cas9 plasmids were generated by replacing the uracil phosphoribosyl transferase (UPRT)-targeting guide ribonucleic acid (gRNA) in pSAG1-Cas9-sg*UPRT* with corresponding gRNAs [9, 16, 17]. All the primers used are presented in Supplementary Table S1. The plasmid was amplified using the Q5 enzyme, ligated into a loop with a ClonExpress II one-step cloning kit (Vazyme Biotech), purified, identified, and then transfected.

The *ΔTgnt2* and *ΔTgnt3* knockout strains were constructed by CRISPR/Cas9-mediated homologous gene replacement. To construct *Tg*NT2- and *Tg*NT3-specific CRISPR plasmids, we replaced the *UPRT*-targeting guide RNA in pSAG1-Cas9-sg*UPRT* with *Tg*NT2 and *Tg*NT3 guide RNAs using Q5 site-directed mutagenesis as described previously. Next, the 5′ and 3′-UTRs (approximately 1 kb) of *Tg*NT2 and *Tg*NT3, as well as dihydrofolate reductase (*DHFR*) (selection markers), were amplified by polymerase chain reaction (PCR) and cloned and inserted into pUC19 using a ClonExpress II One Step Cloning Kit (Vazyme Biotech, Nanjing, China). To construct the *ΔTgnt2* and *ΔTgnt3* strains, the DHFR-*Tg*NT2/*Tg*NT3 amplicon was cotransfected with pSAG1-CAS9-sg*TgNT2/TgNT3* into the RH*Δhxgprt* strain. Pyrimethamine (1 μM, Sigma) was used as the drug for transfectant screening, and single colonies were subsequently screened using the limited dilution method and identified by diagnostic PCR [9, 16].

The i*Tg*NT1 strain containing an *N*-terminal Ty tag (EVHTNQDPLD) was generated by amplifying the coding sequence (CDS) of *Tg*NT1 to replace the *Tg*ANT cassette in pSAG1-TetO7-*iTgANT* [14]. The resulting construct was linearized with upstream and downstream primers, transfected into the TATi strain with 7.5 μg *pSAG1-Cas9-sgTgNT1* plasmid, and allowed to infect fresh HFFs for 24 h post transfection. Clones were isolated by limiting dilution with 1 μM pyrimethamine. Anhydrotetracycline (ATc, TaKaRa) was used to deplete *Tg*NT1 expression in all i*Tg*NT1 strains at a final concentration of 0.5 μg/mL [14, 15, 18]. The complementation and heterologous complementation strains were generated at the *UPRT* locus using the pSAG1-Cas9-sg*UPRT* plasmid as previously described [14].

### Immunofluorometric assay

HFF cells cultured on coverslips were infected and then allowed to grow for 24 h. After that, the parasites were fixed in 4% paraformaldehyde for 15 min and washed in phosphate buffered saline (PBS), followed by permeabilization with 0.1% Triton solution for 20 min and washing in PBS, followed by occlusion with 10% FBS for 25 min at 37 °C. Then, the coverslips were mounted onto microscope slides (Citotest, China) containing antifade mounting medium (Beyotime, China) and imaged under an Olympus FluoView FV1000 confocal microscope (Olympus Life Science, Japan). For the replication assay, the number of parasites per vacuole was counted, with approximately 150 vacuoles evaluated under each coverslip.

The primary antibodies, i.e., mouse anti-HA (Medical & Biological Laboratories Co., Ltd.) [14], rabbit anti-Ty monoclonal antibody (gift from Prof. David Sibley), rabbit anti-ALD (gift from Prof. David Sibley) [19], rabbit anti-Hsp60 (produced in our laboratory) [20], rabbit anti-IMC1 (produced in our laboratory), and rabbit anti-SERCA (produced in our laboratory) [14] were used in this study. Alexa Fluor 488- and Alexa Fluor 594-conjugated goat anti-mouse antibodies were used as secondary antibodies. The cell nuclei were stained with Hoechst 33,342 (Beyotime, C1022).

### Egress assay

Pretreated parasites were collected, counted, and 1 × 10^6^ parasites were used to infect coverslips covered with a HFF monolayer, followed by 36 h of culture. Next, A23187 was added to the cultures to a final concentration of 2 µM, and the cultures were incubated for 2 min to induce parasite egress. Subsequently, the samples were fixed and stained with an anti-GRA7 antibody to assess the integrity of parasitophorous vacuoles (PVs). Egress efficiency was calculated as the percentage of egressed PVs.

### Invasion assay

Fresh tachyzoites were collected by mechanically lysing host cells, then seeded onto HFF monolayer-covered coverslips in 24-well plates at a density of 1 × 10^6^ tachyzoites per well in the presence or absence of ATc. These were incubated at 37 °C for 20 min. Then, 4% paraformaldehyde was added to halt invasion. Extracellular parasites were stained using mouse anti-*T. gondii* antibodies (produced in our laboratory), followed by washing with PBS. The cells were then permeabilized with 0.1% Triton X-100 and stained with the rabbit anti-ALD antibodies. The invasion rate is equal to the number of parasites stained with ALD minus the number of parasites stained with mouse anti-*T. gondii* divided by the number of cell nuclei.

### Plaque assay

Plaque assays were performed using confluent monolayers of HFFs in six-well plates. The cells were infected with 200 *T. gondii* tachyzoites per well in the presence or absence of ATc and incubated undisturbed at 37 °C for 7 days. Following incubation, the HFF monolayers were methanol-fixed and stained with crystal violet. Plaques were enumerated using image analysis software (Adobe Photoshop).

### Statistical analyses and mapping

Protein sequence comparison was performed using BioEdit software [21], protein structure prediction was performed using AlphaFold [22, 23], indirect fluorescent antibody (IFA) data were processed using ZNESS software [14], replication data were statistically analyzed in Excel, replica maps were created using Prism software, and null plots were created using PS software.

### Metabolomic analysis

The samples for the quantitative targeted metabolism assay were divided into two groups: NT1-TATi and NT1-TATi-ATc. Each group consisted of six replicates, with 2 × 10^8^ *T. gondii* collected from each sample and stored at −80 °C. Samples were thawed on ice (subsequent operations were performed on ice). After transfer and washing with normal saline, samples were resuspended by vortexing for 2 min. A total of 50 μL resuspension was transferred to a new tube, and 200 μL precooled (−20 °C) methanol was added, followed by vortexing. Samples were subjected to cycles of freezing in liquid nitrogen (5 min), standing on ice (5 min), and vortexing (2 min) (repeated three times), then centrifuged (10,000*g* for 10 min at 4 °C). Supernatant was collected, incubated at −20 °C for 30 min, recentrifuged, and processed for liquid chromatography–mass spectrometry (LC–MS) analysis.

Sample extracts were analyzed using a liquid chromatography–electrospray ionization–tandem mass spectrometry (LC–ESI–MS/MS) system consisting of an ExionLC AD UPLC and an AB 6500 + QTRAP^®^ mass spectrometer (MS) (Sciex, https://sciex.com.cn/, https://sciex.com/), with two chromatographic methods employed. For the T3 method, separation was performed on a Waters ACQUITY UPLC HSS T3 C18 column (100 mm × 2.1 mm i.d., 1.8 μm) using mobile phases A and B under a gradient of 5% B (0 min) → 95% B (8–9.5 min) → 5% B (9.6–12 min), at a flow rate of 0.35 mL/min, a column temperature of 40 °C, and an injection volume of 2 μL. For the Amide method, an ACQUITY UPLC BEH Amide column (2.1 mm × 100 mm i.d., 1.7 μm) was used with mobile phases A (water with 10 mM ammonium acetate and 0.3% ammonium hydroxide) and B (90:10 acetonitrile:water, *V*/*V*) under a gradient of 95% B (0–1.2 min) → 70% B (8 min) → 50% B (9–11 min) → 95% B (11.1–15 min), at a flow rate of 0.4 mL/min, a column temperature of 40 °C, and an injection volume of 2 μL. For electrospray ionization–tandem mass spectrometry (ESI–MS/MS) detection, the AB 6500 + QTRAP^®^ system was equipped with an electrospray ionization (ESI) Turbo Ion-Spray interface, operated in both positive and negative ion modes, and controlled by Analyst 1.6 software (AB Sciex); key ESI source parameters included turbo spray ion source, source temperature of 550 °C, ion spray voltage (IS) of 5500 V (positive) and −4500 V (negative), and curtain gas (CUR) set at 35.0 psi. Declustering potential (DP) and collision energy (CE) for individual multiple reaction monitoring (MRM) transitions were further optimized, and specific MRM transitions were monitored per period on the basis of the metabolites eluted during that time.

## Results

### *Tg*NTs exhibit different subcellular localizations

**Fig. 1:**
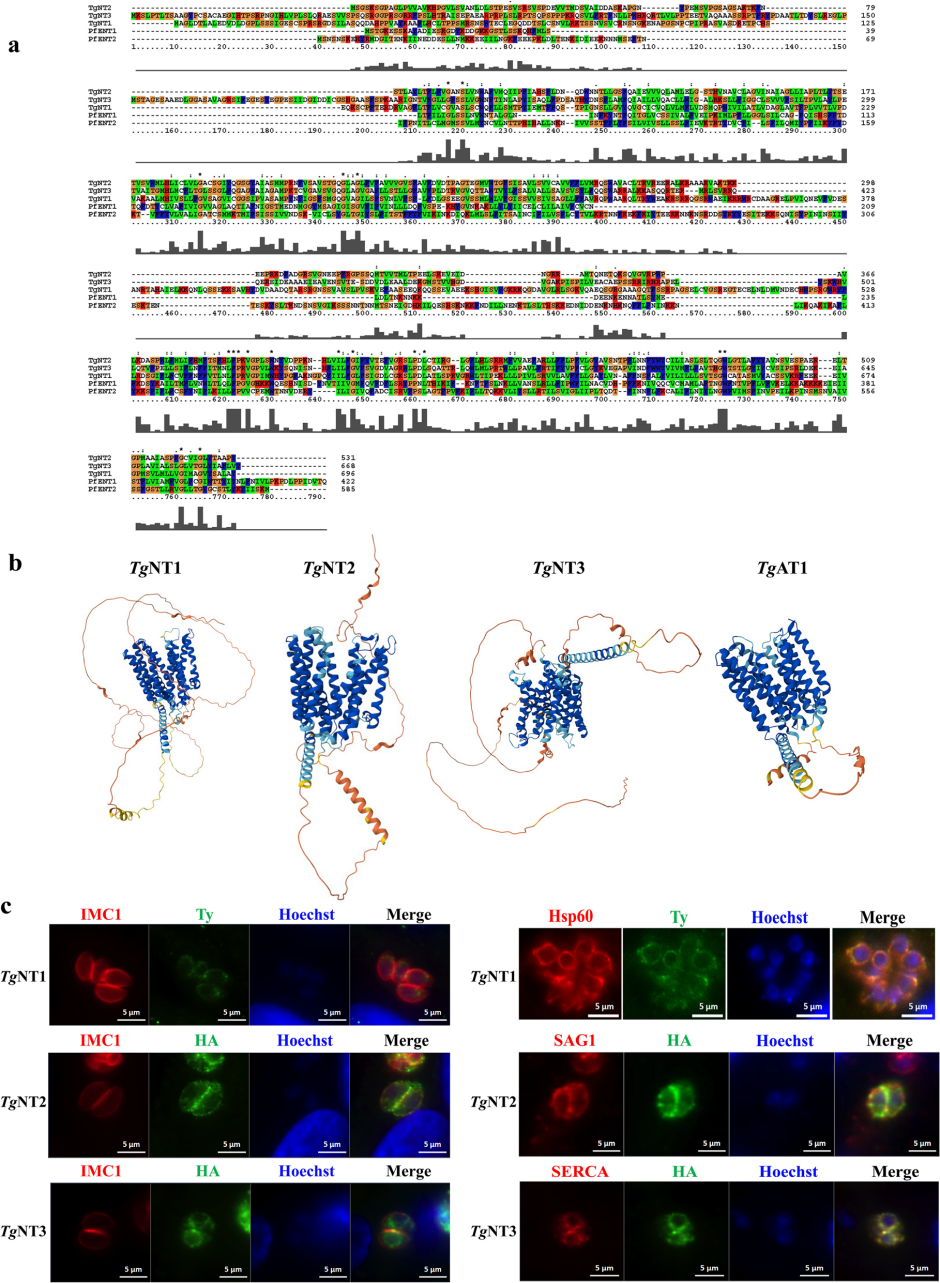
Sequence alignment, structural prediction, and subcellular localization of *Tg*NTs. **a** Protein sequences were downloaded from NCBI with gene ID codes for *P. falciparum* (NT1, gene ID no. 2655415; NT2, gene ID no. 814217). **b** The hypothetical structures of *Tg*NT1, *Tg*NT2, *Tg*NT3, and *Tg*AT1 were downloaded from ToxoDB. **c** *Tg*NT1-Ty immunostaining using anti-Ty with HSP60, *Tg*NT2-HA immunostaining using anti-HA with SAG1, and *Tg*NT3-HA immunostaining using anti-HA with SERCA.

In *T. gondii*, *Tg*AT1 remains the sole identified nucleoside transporter to date. To investigate the potential existence of additional nucleoside transporters in this parasite, we conducted a Basic Local Alignment Search Tool (BLAST) analysis using the amino acid sequence of *Tg*AT1 in the ToxoDB database. This query revealed three candidate nucleoside transporter genes, TGGT1_288540, TGGT1_233130, and TGGT1_359630, which are already annotated as nucleoside transporters in ToxoDB. We designated these proteins *Tg*NT1, *Tg*NT2, and *Tg*NT3, respectively. Notably, they are uncharacterized and require experimental confirmation. Comparison of the amino acid sequences of these nucleoside transporters revealed that there was no homology between the three nucleoside transporters and the host proteins. *Tg*NT1 is a large protein of 696 amino acids in length and is predicted to have 11 transmembrane structural domains, which were found to have some sequence similarity to *Plasmodium falciparum* (*P. falciparum*) nucleoside transporter protein 2 (*Pf*NT2) by sequence comparison, with a similarity of 18.7%, whereas *Tg*NT2 is 531 amino acids in length and is also predicted to have 11 transmembrane structural domains (Supplementary Fig. S1). The sequences of *P. falciparum* nucleoside transporter protein 1 (*Pf*NT1) and *Tg*NT1 exhibited 27.1% similarity (Fig. 1a). In addition, the three-dimensional (3D) structures of *Tg*NT1, *Tg*NT2, *Tg*NT3, and *Tg*AT1 were compared (Fig. 1b). The results indicated that *Tg*NTs share a helical architectural feature with *Tg*AT1. Helices are a prevalent structural element in membrane transporters, typically participating in the formation of transmembrane channels or substrate-binding pockets, which aligns with their common function as nucleoside transporters.

To verify the subcellular location of the proteins, HA tags were fused to the C-termini of *Tg*NT2 and *Tg*NT3, and Ty tags were fused to the N terminus of *Tg*NT1. Immunofluorescence staining revealed that the three proteins had different subcellular locations, with *Tg*NT1 located in the mitochondria, *Tg*NT2 in the plasma membrane, and *Tg*NT3 in the ER (Fig. 1c).

### *Tg*NT1 is essential for parasite growth

**Fig. 2:**
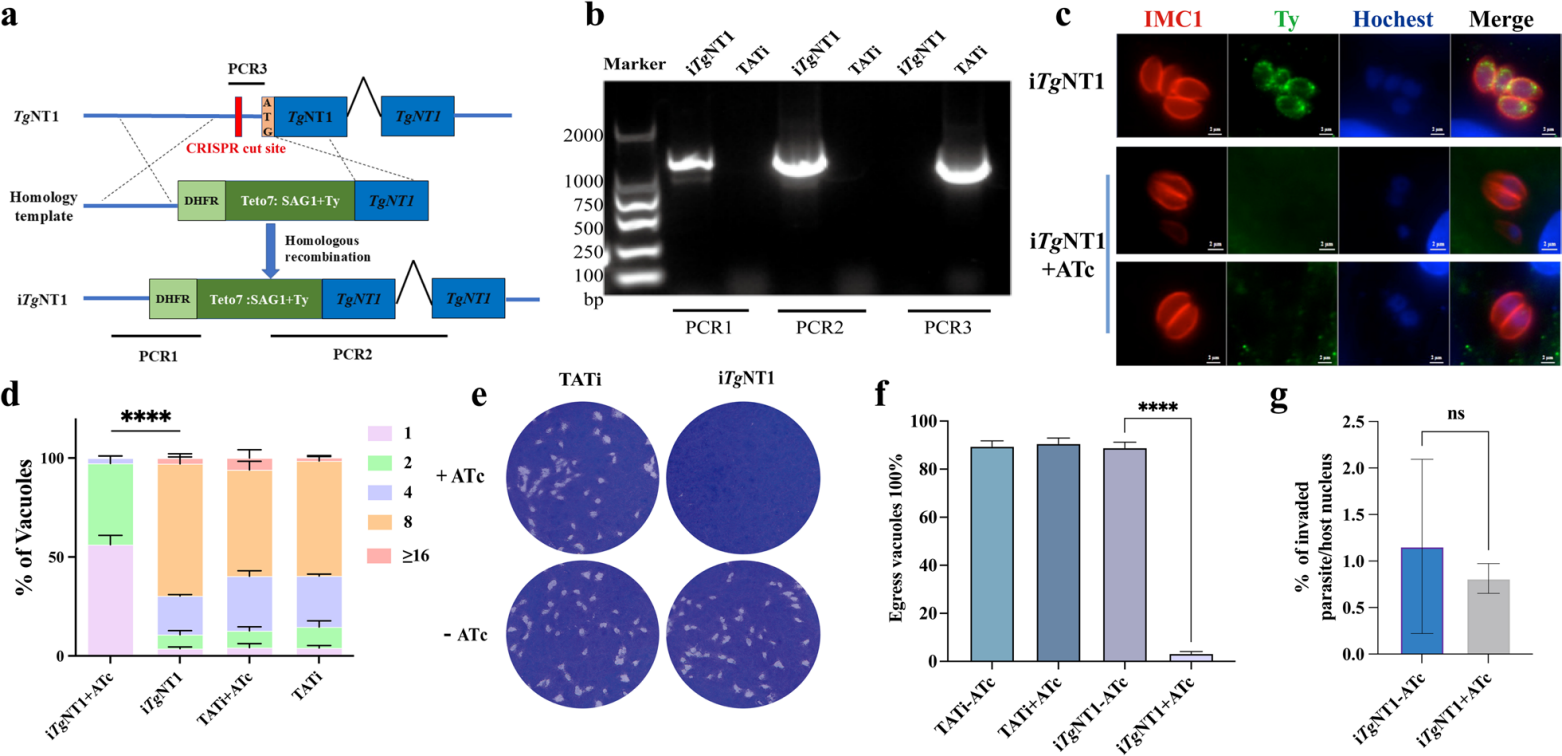
Conditional knockdown of *Tg*NT1 results in severe growth defects. **a** Schematic representation of the conditional knockdown of *Tg*NT1. CRISPR/Cas9 homologous recombination was used to replace the endogenous promoter of *Tg*NT1 with a tetracycline-regulatable promoter (SAG1::Teto7) in the TATi line. **b** PCR assay of i*Tg*NT1, with wild-type TATi strains used as the control group. **c** IFA of i*Tg*NT1 pretreated with or without ATc for 48 h. **d** In vitro replication assay of i*Tg*NT1. i*Tg*NT1 cells were inoculated with a monolayer of HFF cells after 48 h of ATc treatment, and after 24 h of replication the number of parasites in each vacuole was calculated. A representative sample from three independent experiments is shown here. Means ± SDs from two replicates. ^****^P < 0.0001, two-way ANOVA. **e** Plaque assay of i*Tg*NT1, with TATi as a control. Parasites were grown for 7 days to form plaques on HFF monolayers ± ATc. **f** Egress of i*Tg*NT1 pretreated with or without ATc for 48 h. The egressed vacuole ratio was calculated in each coverslip. Means ± SEM of three independent experiments. ^****^P < 0.0001, unpaired Student’s *t*-test. **g** Invasion assay of i*Tg*NT1 following 48-h pretreatment with or without ATc. The mean number of parasites per host cell was calculated on each coverslip. Means ± SD of three independent experiments. Unpaired Student’s *t*-test. *SD* standard deviation, *ANOVA* analysis of variance, *SEM* standard error of the mean

The low phenotypic value (−3.86) of *Tg*NT1 suggests that it is essential for parasite growth [24]. To explore the biological role of *Tg*NT1 in *T. gondii*, the TATi conditional depletion system was used to downregulate *Tg*NT1 expression, where the native *Tg*NT1 promoter was replaced with a tetracycline-dependent promoter. The expression of *Tg*NT1 was downregulated by the addition of ATc, and a Ty epitope was cloned in the N terminus of *Tg*NT1 to detect the depletion of *Tg*NT1. Specific primers verified that the above components were correctly integrated (Fig. 2a, b). Following 48 h of ATc treatment, immunofluorescence assays demonstrated the disappearance of the Ty epitope, confirming the inhibition of i*Tg*NT1 expression (Fig. 2c). The in vitro growth phenotype of i*Tg*NT1 was subsequently evaluated. After treatment with ATc, the intracellular replication speeds of the i*Tg*NT1 strains were significantly reduced (Fig. 2d). In the plaque assay, the i*Tg*NT1 strains treated with ATc failed to form any visible plaques compared with the i*Tg*NT1 strains without ATc treatment (Fig. 2e). In addition, egress defects were detected in the i*Tg*NT1 strains treated with ATc (Fig. 2f). Taken together, these data indicate that *Tg*NT1 is essential for the normal growth of *Toxoplasma*. However, there was no statistically significant difference in invasion ability before and after ATc treatment (Fig. 2g).

**Fig. 3:**
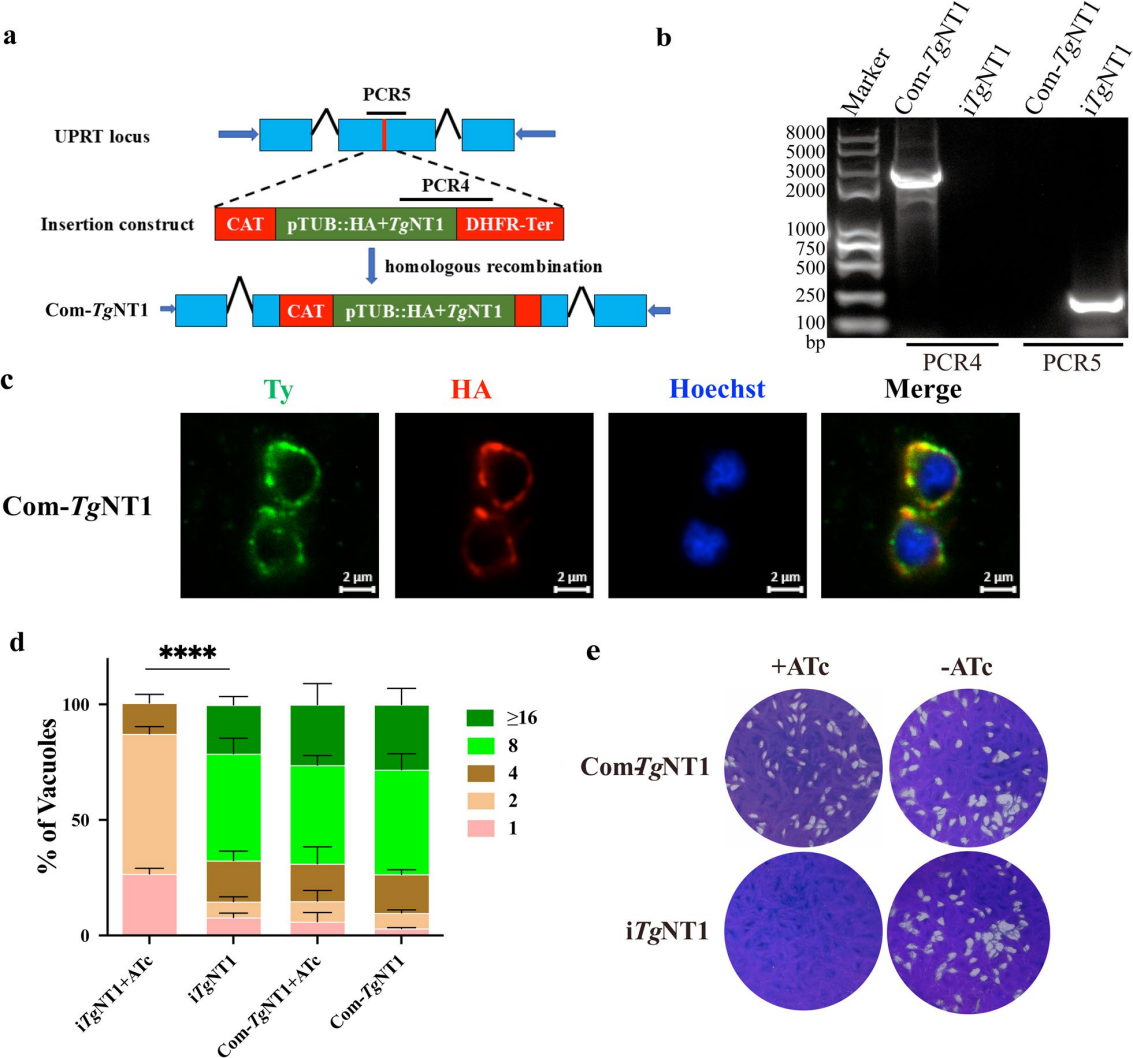
Construction and in vitro phenotyping of the Com-*Tg*NT1 strain. **a** Diagram showing the insertion of NT1 into the *UPRT* site of i*Tg*NT1 using homologous recombination. **b**, **c** PCR and immunofluorescence staining confirmed the successful construction of the complemented strain. The i*Tg*NT1 strain was added as a control (**b**). Immunostaining of Com-*Tg*NT1 with anti-HA and anti-Ty antibodies revealed that both were expressed in mitochondria (**c**). **d**, **e** Detection of the growth speed of Com-*Tg*NT1 in a replication assay (**d**) and a plaque assay (**e**). ^****^P < 0.0001, two-way ANOVA

To verify that specific growth defects are caused by i*Tg*NT1, the full-length CDS of *Tg*NT1, which was fused with a HA tag, was recombined into the UPRT locus with i*Tg*NT1 (Fig. 3a). PCR revealed that the CDS was correctly integrated into the *UPRT* gene, and IFA confirmed the expression and localization of *Tg*NT1 to the mitochondria (Fig. 3b, c). There was no discernible difference in plaque formation or replication in the Com-*Tg*NT1 strain with or without ATc treatment (Fig. 3d, e). This is in stark contrast to the i*Tg*NT1 growth phenotype. Overall, *Tg*NT1 is essential for the growth of tachyzoites in vitro.

### *Tg*NT2 and *Tg*NT3 are nonessential for *T. gondii* tachyzoite growth

**Fig. 4:**
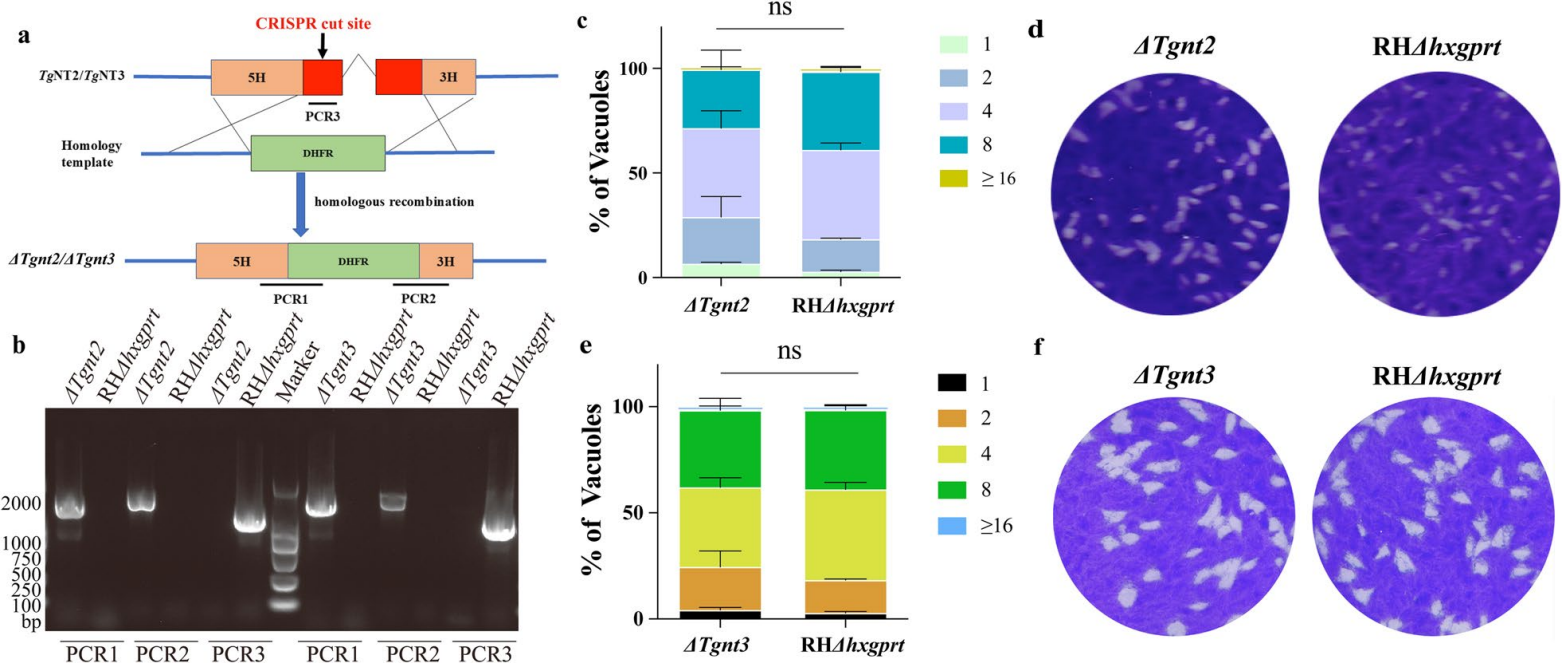
Construction and in vitro phenotyping of *ΔTgnt2* and *ΔTgnt3*. **a** On the basis of phenotype scores, *Tg*NT2 and *Tg*NT3 were predicted to be dispensable [24] following the construction of direct knockout strains by replacing the target gene with DHFR. **b** PCR was used to verify successful insertion of DHFR into *ΔTgnt2* and *ΔTgnt3*, with RH*Δhxgprt* used as a control. **c**, **d** The in vitro phenotypes of the *ΔTgnt2* strains were not defective, with no differences in the replication assay (**c**) or plaque assay (**d**) results compared with those of the RH*Δhxgprt* strains. **e**, **f** The in vitro phenotypes of the *ΔTgnt3* strains were not defective, with no plaque differences in (**e**) or (**f**) compared with those of the RH*Δhxgprt* strains

We also evaluated the physiological importance of *Tg*NT2 and *Tg*NT3. CRISPR/Cas9-mediated genome editing technology was used to knock out *NT2* and *NT3* in the RH*Δhxgprt* strain directly (Fig. 4a). PCR validation was performed on the deletion strains obtained through DHFR-mediated drug screening (Fig. 4b). In vitro growth assays revealed that the deletion strains did not significantly differ from RH*Δhxgprt* in either replication or plaque assays (Fig. 4c–f). This finding indicated that the deletion of *NT2* and *NT3* did not affect the in vitro growth of tachyzoites.

### Nucleoside analog alterations in i*Tg*NT1 elucidate its phenotypic defects

**Fig. 5:**
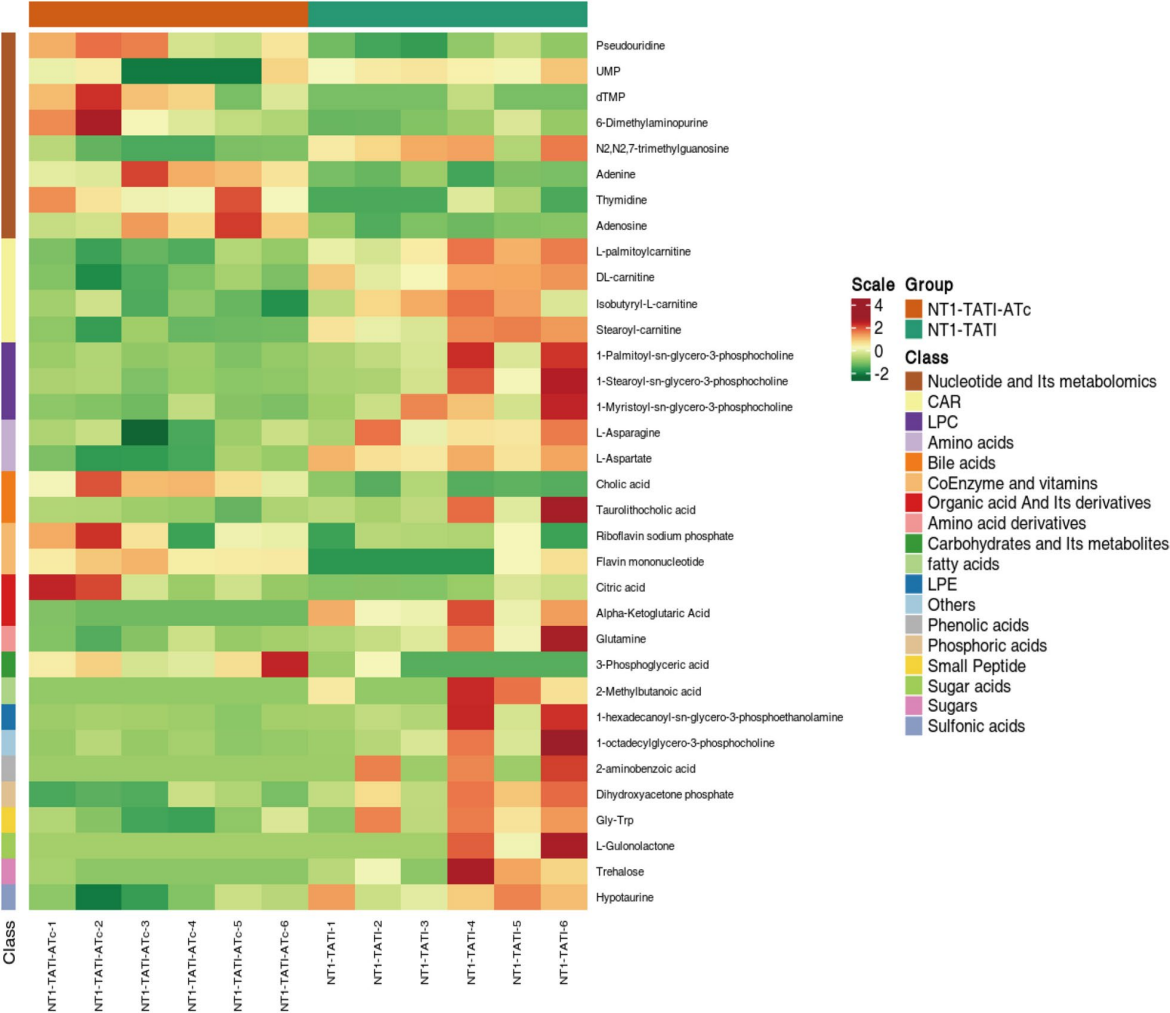
Heatmap of differentially abundant metabolites. Many different types of metabolites, such as nucleosides, carnitines, lipids, and amino acids, are altered

To further elucidate the molecular basis underlying the phenotypic defects of *Tg*NT1 depletion mutants, we determined the levels of metabolites related to cellular metabolism in i*Tg*NT1 parasites via LC–MS/MS. A total of 253 metabolites were detected in the samples (Supplementary Table S2). By analyzing metabolite abundance, we identified 34 metabolites with altered levels in i*Tg*NT1 after ATc treatment—compared with those in i*Tg*NT1 not treated with ATc: 23 were upregulated, whereas 11 were downregulated (Table 1). A clustered heatmap analysis was performed to visualize all differentially expressed metabolites, revealing that nucleosides exhibited the most prominent differences. Among these significantly altered metabolites, increased levels were detected for dTMP, 6-dimethylaminopurine, adenine, adenosine, and thymine, whereas decreased levels were observed for UMP and *N*2,*N*2,7-trimethylguanosine (Fig. 5). We further analyzed the functions of these metabolites in relevant metabolic pathways, which may facilitate the identification of the underlying cause of phenotypic abnormalities in the *Tg*NT1 depletion strain. Beyond the affected nucleoside metabolism, notable alterations were also observed in key energy metabolic pathways. Analysis of the tricarboxylic acid (TCA) cycle showed increased citric acid levels and decreased α-ketoglutarate levels. In addition, metabolite changes were detected in the glycerophosphate metabolic pathway: dihydroxyacetone phosphate and sn-glycerol-3-phosphate—two metabolites produced by this pathway that also participate in gluconeogenesis—were reduced. Furthermore, aberrant levels were observed for some amino acids involved in aminoacyl transfer RNA (tRNA) synthesis, with decreased levels of glutamine, aspartic acid, and aspartame.

**Table 1:** Statistics of the differentially abundant metabolite data

Group name	Total significantly different	Downregulated	Upregulated
NT1-TATi-ATc versus NT1-TATi	34	23	11

## Discussion

It is generally accepted that nucleoside transporter proteins located on the surface of protozoan cell membranes play crucial roles. However, few studies have been conducted on nucleoside transporter proteins on the organelle membranes of protozoa. In this study, the biological characterization of three putative *T. gondii* nucleoside transporter proteins revealed that nucleoside transporter proteins located on the mitochondrion are indispensable for the in vitro growth of *T. gondii* tachyzoites. Previous studies have demonstrated that humans also possess a nucleoside transporter localized to organelle membranes—the human lysosomal nucleoside transporter. Its deficiency leads to severe congenital rare diseases in humans and impairs nucleoside transport between lysosomes and ER [25, 26]. These findings indicate that nucleoside transporters localized to organelle membranes may play a more significant role than previously documented [27, 28]. *Tg*NT1, *Tg*NT2, and *Tg*NT3 were annotated as nucleotide transporters in *ToxoDB*, but their substrate affinities have not been verified by further experiments. Some researchers have proposed that one of these transporters encodes the protein responsible for the nucleoside transport activity of *Tg*AT2 or *Tg*NBT1, though this has yet to be determined [29]. Besides, recent studies have shown that *Tg*NT1 and *Tg*NT2 are also related to the regulation of Ca^2+^ homeostasis in *T. gondii* and the signal cascade of AGC kinases [30, 31].

Quantitative analysis of differentially abundant metabolites, based on LC–MS/MS, revealed that the levels of 34 metabolites were altered following depletion of the *Tg*NT1 protein, with the most significant changes observed in nucleosides. The results demonstrated that the levels of adenosine, adenine, and thymine were elevated. The cell membrane of *T. gondii* contains at least two adenosine transporter proteins that can maintain the demand for adenosine metabolism [10, 11]. Since the uptake of host-derived adenosine via plasma membrane transporters in *T. gondii* remained unaffected by *Tg*NT1 depletion, we hypothesize that the observed accumulation of adenosine and adenine in i*Tg*NT1 strains likely stems from impaired mitochondrial supply and utilization. Pseudouridine is involved in the modification of various RNAs. Studies on pathogenic microorganisms have shown that pseudouridine can facilitate host immune evasion via messenger RNA (mRNA) modification [32, 33]. Under certain pathological conditions, elevated pseudouridine levels in cells may stem from increased conversion rates of specific tRNAs, as well as qualitative and quantitative changes in tRNA-modifying enzymes. These alterations induce changes in cellular metabolism, which in turn lead to morphological modifications. The elevated levels of pseudouridine indicate that the metabolic and replication abnormalities observed in *T. gondii* lacking *Tg*NT1 are a contributing factor to its inability to sustain growth in vitro.

Carnitines are another major class of substances that undergo changes subsequent to nucleosides. Studies have shown that carnitines can facilitate the transport of fats into mitochondria for β-oxidation. After lacking *Tg*NT1, the content of all differential carnitines decreased, indicating that the fatty-acid-oxidation process in the parasite may be inhibited [34]. Furthermore, the content of α-ketoglutarate—a tricarboxylic-acid-cycle intermediate—decreased, as did that of dihydroxyacetone phosphate, which is involved in glucose glycolysis. These observed metabolic alterations suggest abnormalities in energy metabolic pathways, which may further exacerbate the in vitro phenotypic abnormalities of *T. gondii*. Choline, a B-vitamin compound, is generated with the facilitation of sn-glycerol-3-phosphate choline; reduced levels of the latter may lead to a B-vitamin deficiency in *T. gondii*. In addition, the levels of glutamine, aspartic acid, and asparagine—amino acids involved in aminoacyl-tRNA synthesis—were all decreased, suggesting that protein synthesis was inhibited. Furthermore, abnormal levels of the nucleoside analogs UMP and dTMP may have induced abnormalities in the synthesis of DNA and RNA in *T. gondii*. These findings suggest that gene expression in *Tg*NT1-deficient parasites may be inhibited.

This study is the first to characterize the biological properties of intracellular nucleoside transporters in *T. gondii* and to elucidate the underlying mechanisms by which depletion of *Tg*NT1 leads to in vitro growth defects. By demonstrating the metabolic alterations induced by such depletion, this work provides a reference for research on intracellular nucleoside transporters in protozoa. While our current findings offer some insights, several limitations still exist. Owing to technical limitations, we were unable to directly validate the functions and substrate specificities of these three transporters using either isotopic tracing or *Xenopus* oocyte expression systems—this constitutes a key focus for future studies.

## Conclusions

Overall, we identified three novel nucleoside transporters in *T. gondii*, namely, *Tg*NT1, *Tg*NT2, and *Tg*NT3, which localize to the mitochondria, plasma membrane, and ER, respectively. Among these, *Tg*NT1 is crucial for the in vitro growth and proliferation of *T. gondii*, whereas *Tg*NT2 and *Tg*NT3 are dispensable. Conditional depletion of *Tg*NT1 impairs multiple metabolic pathways in both mitochondria and cells, and the most prominent changes manifest in the levels of various nucleosides. Given that *Tg*NT1 has no mammalian homologs, it represents a promising drug target.

## Supplementary Information

Supplementary material 1.

Supplementary material 2. Table S1. Primers used in this study.

Supplementary material 3. Table S2. A total of 253 metabolites’ relative abundances were detected in the sample.

## Data Availability

All data generated or analyzed during this study are included in this published article and its supplementary information files.

## Abbreviations

*T. gondii*: *Toxoplasma gondii*
AT: Adenosine transporter
NT: Nucleoside transporter protein
GPI: Glycosylphosphatidylinositol
AIDS: Acquired immunodeficiency syndrome
ER: Endoplasmic reticulum
HFF: Human foreskin fibroblasts
DMEM: Dulbecco’s modified Eagle medium
FBS: Fetal bovine serum
gRNA: Guide RNA
UPRT: Uracil phosphoribosyl transferase
DHFR: Dihydrofolate reductase
ATc: Anhydrotetracycline
PVs: Parasitophorous vacuoles
ALD: Fructose-1-phosphate aldolase
IMC1: Inner membrane complex protein 1
HSP60: Heat shock protein 60
SERCA: Sarco/endoplasmic reticulum Ca^2+^-ATPase
SAG1: Surface antigen 1
GRA7: Dense granule protein 7
*P. falciparum*: *Plasmodium falciparum*
LC–MS/MS: Liquid chromatography–tandem mass spectrometry

## References

[1] Hurt K, Kodym P, Stejskal D, Zikan M, Mojhova M, Rakovic J. Toxoplasmosis impact on prematurity and low birth weight. PLoS ONE. 2022;17:e0262593. 10.1371/journal.pone.0262593. 35025961 10.1371/journal.pone.0262593 PMC8758008

[2] Montoya JG, Remington JS. Management of *Toxoplasma gondii* infection during pregnancy. Clin Infect Dis. 2008;47:554–66. 10.1086/590149. 18624630 10.1086/590149

[3] Hajimohammadi B, Ahmadian S, Firoozi Z, Askari M, Mohammadi M, Eslami G, et al. A meta-analysis of the prevalence of toxoplasmosis in livestock and poultry worldwide. EcoHealth. 2022;19:55–74. 10.1007/s10393-022-01575-x. 35133541 10.1007/s10393-022-01575-x PMC8823692

[4] Elsheikha HM. Congenital toxoplasmosis: priorities for further health promotion action. Public Health. 2008;122:335–53. 10.1016/j.puhe.2007.08.009. 17964621 10.1016/j.puhe.2007.08.009

[5] Dunay IR, Gajurel K, Dhakal R, Liesenfeld O, Montoya JG. Treatment of toxoplasmosis: Historical perspective, animalmodels and current clinical practice. Clin Microbiol Rev. 2018;31(4): e00057-17. 10.1128/CMR.00057-17 PMC6148195 30209035

[6] Elati HAA, Goerner AL, Martorelli Di Genova B, Sheiner L, de Koning HP. Pyrimidine salvage in *Toxoplasma gondii* as a target for new treatment. Front Cell Infect Microbiol. 2023;13:1320160. 10.3389/fcimb.2023.1320160. 38162577 10.3389/fcimb.2023.1320160 PMC10755004

[7] Montazeri M, Mehrzadi S, Sharif M, Sarvi S, Tanzifi A, Aghayan SA, et al. Drug resistance in *Toxoplasma gondii*. Front Microbiol. 2018;9:2587. 10.3389/fmicb.2018.02587. 30420849 10.3389/fmicb.2018.02587 PMC6215853

[8] Gupte A, Buolamwini JK, Yadav V, Chu CK, Naguib FN, el Kouni MH. 6-Benzylthioinosine analogues: promising anti-toxoplasmic agents as inhibitors of the mammalian nucleoside transporter ENT1 (es). Biochem Pharmacol. 2005;71:69–73. 10.1016/j.bcp.2005.10.031. 16310172 10.1016/j.bcp.2005.10.031

[9] Xia N, Ye S, Liang X, Chen P, Zhou Y, Fang R, et al. Pyruvate homeostasis as a determinant of parasite growth and metabolic plasticity in *Toxoplasma gondii*. MBio. 2019;10:e00898–19. 10.1128/mBio.00898-19. 31186321 10.1128/mBio.00898-19 PMC6561023

[10] De Koning HP, Al-Salabi MI, Cohen AM, Coombs GH, Wastling JM. Identification and characterisation of high affinity nucleoside and nucleobase transporters in *Toxoplasma gondii*. Int J Parasitol. 2003;33:821–31. 10.1016/s0020-7519(03)00091-2. 12865082 10.1016/s0020-7519(03)00091-2

[11] Chiang CW, Carter N, Sullivan WJ Jr, Donald RG, Roos DS, Naguib FN, et al. The adenosine transporter of *Toxoplasma gondii*. Identification by insertional mutagenesis, cloning, and recombinant expression. J Biol Chem. 1999;274:35255–61. 10.1074/jbc.274.49.35255. 10575012 10.1074/jbc.274.49.35255

[12] Schwab JC, Afifi Afifi M, Pizzorno G, Handschumacher RE, Joiner KA. *Toxoplasma gondii* tachyzoites possess an unusual plasma membrane adenosine transporter. Mol Biochem Parasitol. 1995;70:59–69. 10.1016/0166-6851(95)00005-l. 7637715 10.1016/0166-6851(95)00005-l

[13] Meissner M, Schlüter D, Soldati D. Role of *Toxoplasma gondii* myosin A in powering parasite gliding and host cell invasion. Science. 2002;298:837–40. 10.1126/science.1074553. 12399593 10.1126/science.1074553

[14] Li S, Qian J, Xu M, Yang J, He Z, Zhao T, et al. A new adenine nucleotide transporter located in the ER is essential for maintaining the growth of *Toxoplasma gondii*. PLoS Pathog. 2022;18:e1010665. 10.1371/journal.ppat.1010665. 35788770 10.1371/journal.ppat.1010665 PMC9286291

[15] Qian J, Zhao T, Guo L, Li S, He Z, He M, et al. Mitochondrial ADP/ATP carrier 1 is important for the growth of *Toxoplasma* tachyzoites. Microbiol Spectr. 2023;11:e0004023. 10.1128/spectrum.00040-23. 37154708 10.1128/spectrum.00040-23 PMC10269819

[16] Shen B, Buguliskis JS, Lee TD, Sibley LD. Functional analysis of rhomboid proteases during *Toxoplasma* invasion. MBio. 2014;5:e01795-e1814. 10.1128/mBio.01795-14. 25336455 10.1128/mBio.01795-14 PMC4212836

[17] Shen B, Sibley LD. *Toxoplasma* aldolase is required for metabolism but dispensable for host-cell invasion. Proc Natl Acad Sci U S A. 2014;111:3567–72. 10.1073/pnas.1315156111. 24550496 10.1073/pnas.1315156111 PMC3948255

[18] Toursel C, Dzierszinski F, Bernigaud A, Mortuaire M, Tomavo S. Molecular cloning, organellar targeting and developmental expression of mitochondrial chaperone HSP60 in *Toxoplasma gondii*. Mol Biochem Parasitol. 2000;111:319–32. 10.1016/s0166-6851(00)00324-8. 11163440 10.1016/s0166-6851(00)00324-8

[19] Li Y, Niu Z, Yang J, Yang X, Chen Y, Li Y, et al. Rapid metabolic reprogramming mediated by the AMP-activated protein kinase during the lytic cycle of *Toxoplasma gondii*. Nat Commun. 2023;14:422. 10.1038/s41467-023-36084-0. 36702847 10.1038/s41467-023-36084-0 PMC9880002

[20] Chen P, Chen Y, Xia N, Fan B, Niu Z, He Z, et al. A pyruvate transporter in the apicoplast of apicomplexan parasites. Proc Natl Acad Sci U S A. 2024;121(25): e2314314121 10.1073/pnas.2314314121 PMC11194499 38865262

[21] Mtshali PS, Mtshali MS. Genetic Characterization and phylogenetic analysis of *Babesia bigemina* isolates incattle from South Africa based on BgRAP-1, BgAMA-1 and BgβTUB Genes. Biology (Basel). 2025;14(4): 355. 10.3390/biology14040355 PMC12025327 40282220

[22] Varadi M, Anyango S, Deshpande M, Nair S, Natassia C, Yordanova G, et al. AlphaFold protein structure database: massively expanding the structural coverage of protein-sequence space with high-accuracy models. Nucleic Acids Res. 2022;50:D439–44. 10.1093/nar/gkab1061. 34791371 10.1093/nar/gkab1061 PMC8728224

[23] Abramson J, Adler J, Dunger J, Evans R, Green T, Pritzel A, et al. Accurate structure prediction of biomolecular interactions with AlphaFold 3. Nature. 2024;630:493–500. 10.1038/s41586-024-07487-w. 38718835 10.1038/s41586-024-07487-w PMC11168924

[24] Sidik SM, Huet D, Ganesan SM, Huynh MH, Wang T, Nasamu AS, et al. A genome-wide CRISPR screen in *Toxoplasma* identifies essential Apicomplexan genes. Cell. 2016;166:1423-35.e12. 10.1016/j.cell.2016.08.019. 27594426 10.1016/j.cell.2016.08.019 PMC5017925

[25] Baldwin SA, Yao SY, Hyde RJ, Ng AM, Foppolo S, Barnes K, et al. Functional characterization of novel human and mouse equilibrative nucleoside transporters (hENT3 and mENT3) located in intracellular membranes. J Biol Chem. 2005;280:15880–7. 10.1074/jbc.M414337200. 15701636 10.1074/jbc.M414337200

[26] Cliffe ST, Kramer JM, Hussain K, Robben JH, de Jong EK, de Brouwer AP, et al. SLC29A3 gene is mutated in pigmented hypertrichosis with insulin-dependent diabetes mellitus syndrome and interacts with the insulin signaling pathway. Hum Mol Genet. 2009;18:2257–65. 10.1093/hmg/ddp161. 19336477 10.1093/hmg/ddp161

[27] Ma H, Qu J, Liao Y, Liu L, Yan M, Wei Y, et al. Equilibrative nucleotide transporter ENT3 (SLC29A3): a unique transporter for inherited disorders and cancers. Exp Cell Res. 2024;434:113892. 10.1016/j.yexcr.2023.113892. 38104646 10.1016/j.yexcr.2023.113892

[28] Bakhchane A, Kindil Z, Charoute H, Benchikhi K, Khadir K, Nadifi S, et al. Compound heterozygous SLC29A3 mutation causes H syndrome in a Moroccan patient: a case report. Curr Res Transl Med. 2016;64:65–8. 10.1016/j.retram.2016.01.008. 27316388 10.1016/j.retram.2016.01.008

[29] Piro F, Focaia R, Dou Z, Masci S, Smith D, Di Cristina M. An uninvited seat at the dinner table: How apicomplexanparasites scavenge nutrients from the host. Microorganisms. 2021;9(12): 2592. 10.3390/microorganisms9122592 PMC8707601 34946193

[30] Herneisen AL, Peters ML, Smith TA, Shortt E, Lourido S. SPARK regulates AGC kinases central to the *Toxoplasma gondii* asexual cycle. Elife. 2024;13:RP93877. 10.7554/eLife.93877. 39136687 10.7554/eLife.93877 PMC11321763

[31] Herneisen AL, Li ZH, Chan AW, Moreno SNJ, Lourido S. Temporal and thermal profiling of the *Toxoplasma* proteome implicates parasite protein phosphatase 1 in the regulation of Ca(^2+^)-responsive pathways. Elife. 2022;11:e80336. 10.7554/eLife.80336. 35976251 10.7554/eLife.80336 PMC9436416

[32] Cui L, Ma R, Cai J, Guo C, Chen Z, Yao L, et al. RNA modifications: importance in immune cell biology and related diseases. Signal Transduct Target Ther. 2022;7:334. 10.1038/s41392-022-01175-9. 36138023 10.1038/s41392-022-01175-9 PMC9499983

[33] Ulland TK, Janowski AM, Buchan BW, Faron M, Cassel SL, Jones BD, et al. *Francisella tularensis* live vaccine strain folate metabolism and pseudouridine synthase gene mutants modulate macrophage caspase-1 activation. Infect Immun. 2013;81:201–8. 10.1128/iai.00991-12. 23115038 10.1128/IAI.00991-12 PMC3536133

[34] Tamai I, Ohashi R, Nezu J, Yabuuchi H, Oku A, Shimane M, et al. Molecular and functional identification of sodium ion-dependent, high affinity human carnitine transporter OCTN2. J Biol Chem. 1998;273:20378–82. 10.1074/jbc.273.32.20378. 9685390 10.1074/jbc.273.32.20378

