# Supplementary figures and images for: A nucleoside transporter on the mitochondria of T. gondii is essential for maintaining normal growth of the parasite
Source: Parasit Vectors. 2025 Oct 21;18:418. doi: 10.1186/s13071-025-07054-w (PMC12538992; doi:10.1186/s13071-025-07054-w)

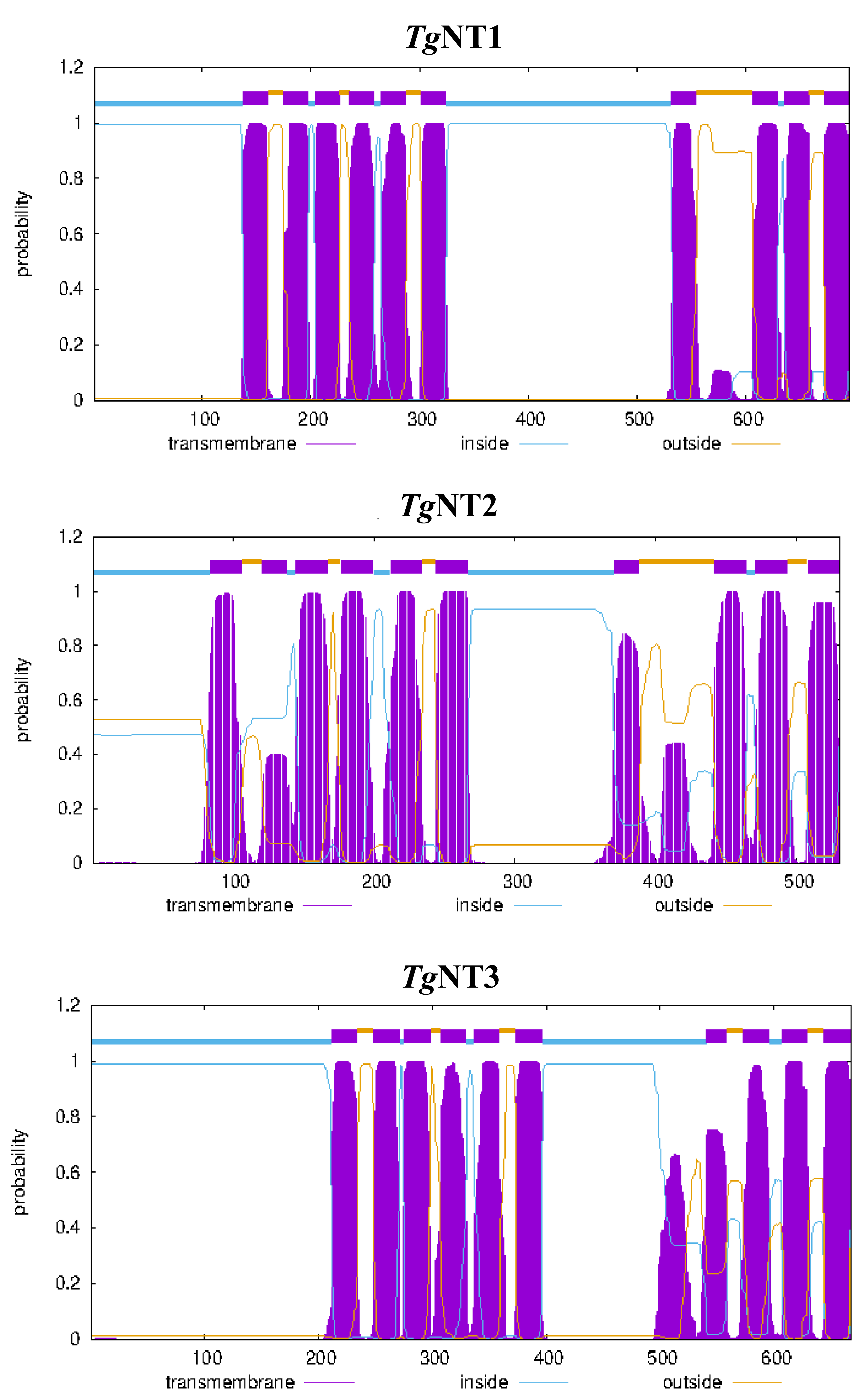

Supplement: Supplementary file 1 — Supplementary material 1. [file 13071_2025_7054_MOESM1_ESM.tif]
